# Endocrine Adverse Events of Nivolumab in Non-Small Cell Lung Cancer Patients—Literature Review

**DOI:** 10.3390/cancers12082314

**Published:** 2020-08-17

**Authors:** Marta Dudzińska, Michał Szczyrek, Kamila Wojas-Krawczyk, Joanna Świrska, Izabela Chmielewska, Agnieszka Zwolak

**Affiliations:** 1 Chair of Internal Medicine and Department of Internal Medicine in Nursing, Medical University of Lublin, 20-093 Lublin, Poland; kardelka@wp.pl (J.Ś.); zwolakag@wp.pl (A.Z.); 2 Department of Pneumology, Oncology and Allergology, Medical University of Lublin, 20-090 Lublin, Poland; mszczyr@gmail.com (M.S.); kamilawojas@wp.pl (K.W.-K.); izachm@wp.pl (I.C.)

**Keywords:** nivolumab, immune-related adverse event, checkpoint inhibitor, non-small cell lung cancer, antithyroid antibody

## Abstract

In recent years, we have observed significant progress in cancer treatment associated with the development of immunotherapy. A programmed cell death 1 molecule (PD-1) on the surface of T lymphocytes may be stimulated via a specific PD-ligand 1 (PD-L1), which inhibits lymphocyte activation and leads to apoptosis. Some malignant cells are characterized by high PD-L1 expression. Nivolumab, an anti-PD-1 antibody, blocks the interaction between PD-1 and its ligands and inhibits the signaling pathway by preventing the tumor-derived PD-L1 from blocking T lymphocytes. In patients with non-small cell lung cancer (NSCLC), it is used either in monotherapy or in combination with other drugs. Immunotherapy is associated with the possibility of immune-related adverse effects (irAE) including endocrinopathies (3–23%). Thyroid disorders are the most common, with severity rarely exceeding grade 2. Hypophysitis, adrenal insufficiency and diabetes are possible complications which require immediate treatment. Individuals with autoimmune diseases diagnosed prior to immunotherapy are at risk of its exacerbation. In the management of patients receiving immunotherapy, evaluation of history of autoimmune diseases, awareness and early diagnosis of irAE are crucial and may affect treatment outcomes.

## 1. Introduction

Recently, we have witnessed the dynamic development of new methods of anti-cancer treatment. One of these is immunotherapy, which works by strengthening the patient’s immune system’s response to cancer cells [1]. This was possible because of our better understanding of the biology of cancer and immunological checkpoints [1].

The function of the immune system is to recognize and tolerate what is “ours” and fight what is “foreign”. The development of cancer is evidence of imperfection in the function of the immune system, which has allowed malignant cells with abnormal properties to multiply out of control.

On the surfaces of T lymphocytes (cells that play a key role in coordinating the course of the immune response), there are molecules that regulate their activity and are involved in maintaining the balance between control of pathogenic microorganisms and excessive activation of lymphocytes that threaten healthy tissue. The so-called immune checkpoints include the following:

1. Activating molecules (CD28, CD27, OX40, CD137 and GITR (glucocorticoid-induced tumor necrosis factor receptor)), whose stimulation increases the proliferation, differentiation and activation of lymphocytes, positively stimulating the immune response;
2. Inhibitory molecules (CTLA-4 (cytotoxic T-limphocyte-associated protein 4), PD-1 (programmed cell death-1), ICOS (inducible T-cell co-stimulator) and LAG-3 (lymphocyte activation gene-3)), the stimulation of which causes the functional depletion of lymphocytes, thus limiting T lymphocyte activity and causing immunosuppression [1].

## 2. Inhibitory Molecules and Their Use in Oncology

The PD-1 molecule (also known as CD279) [2], a programmed cell death 1 molecule, is primarily found on the surfaces of activated T lymphocytes, as well as NK (natural killer) cells, thymocytes and active B lymphocytes [3,4]. In chronic inflammation, it is also expressed on macrophages, monocytes and dendritic cells [4]. PD-1 is a transmembrane glycoprotein weighing 50–55 kDa [5,6]. When a T lymphocyte is activated in cooperation with an antigen-presenting cell, the PD-1 molecule is expressed on the cell surface and interferon production rises, which in turn stimulates the expression of the PD-L1 molecule in tissue [5]. PD-1 receptor stimulation via specific PD-L1 (also known as B7-H1 or CD274) and/or PD-L2 (also known as B7-DV or CD273) ligands [2] inhibits the cascade of kinases involved in lymphocyte activation and leads to lymphocyte apoptosis by the expression of genes responsible for programmed cell death. The proliferation and differentiation of T lymphocytes and cytokine production are also inhibited, causing the inhibition of antibody production by B lymphocytes. The interaction of PD-1 and PD-L1 leads to the phosphorylation of the PD-1 intracellular tyrosine domain, which in turn leads to glucose metabolism quenching, a decrease in protein synthesis and the production of interleukin-10, which is a key factor inducing the anergy of CD4 + and CD8 + T lymphocytes [3]. In the case of other immune cells, stimulation of the PD-1 molecule results in inhibition of antibody production in B lymphocytes and lytic activity of NK cells [3,4,7]. As a result, immune response is suppressed.

The PD-L1 molecule is found on antigen-presenting cells in lymphoid tissue, dendritic cells and macrophages [1,2], various epithelial and endothelial cells, as well as on the cell surface in immunologically privileged tissues including the retina, Langerhans island or placenta. Particularly intense expression of the PD-L1 molecule occurs from the fourth month of pregnancy. The expression of PD-L1 has also been reported in the cornea and the iris-ciliary body. In a state of immunological balance, PD-L1 expression is low, but this increases in the event of inflammation. PD-L1 is widely expressed in cells of the thymus—the organ supervising central immunotolerance. Therefore, the PD-1/PD-L1 signaling system contributes to the maturation of immunocompetent cells [3]. PD-L2 is expressed mainly on antigen-presenting cells in lung tissue [8].

In contrast to the CTLA-4 molecule, which regulates the activation of T lymphocytes at an early stage of the immune response, inhibiting their autoreactivity within the lymph nodes, in the case of an immune response involving PD-1 molecules, this inhibition occurs in peripheral tissue or in the tumor microenvironment [9].

In the context of the development of the proliferative process, cells of some tumors are characterized by high PD-L1 expression (these include melanoma, non-small cell lung cancer, some lymphomas and breast, ovarian, bladder and kidney cancer) [6], which results in the inhibition of lymphocytes infiltrating cancerous tissue, impacting the body’s natural defense mechanism [1].

The possibility of influencing this mechanism has opened up a new avenue of work for pharmacotherapy in oncology. In 2018, Professors James P. Allison and Tasuku Honjo were honored with the Nobel Prize in medicine and physiology for their research on CTLA-4 and PD-1 particles [10,11,12]. The first drug that targeted the mechanism of immune control checkpoints was ipilimumab—a monoclonal anti-CTLA-4 antibody—which was successfully used to treat metastatic melanoma.

**Figure 1:**
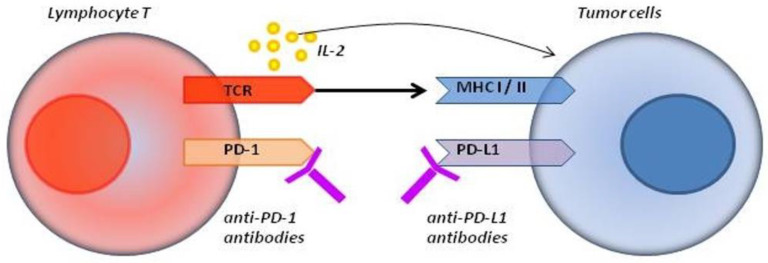
The blocking of programmed cell death 1 (PD-1) molecules (with anti-PD-1 antibodies) or PD-L1 molecules (with anti-PD-L1 antibodies) restores the cytotoxic function of T lymphocytes [15,16].

Currently used immune checkpoint inhibitors (ICI), apart from ipilimumab, include the anti-PD-1 antibodies nivolumab and pembrolizumab and anti-PD-L1 antibodies atezolizumab, durvalumab and avelumab [9]. Antibodies that attach to the PD-1 receptor block the interaction between PD-1 and its ligands PD-L1 and PD-L2 and inhibit the signaling pathway by preventing tumor-derived PD-L1 from blocking T lymphocytes [13]. Nivolumab and pembrolizumab thus restore the natural capabilities of the immune system by increasing the activity of lymphocytes in the tumor microenvironment as well as in peripheral tissue [1,14]. The mechanism of action is presented in Figure 1.

## 3. Adverse Events Associated with Anti-PD-1 and Anti-PD-L1 Immunotherapy

Nivolumab is used in monotherapy or in combination with ipilimumab, among others, in patients with advanced melanoma and locally advanced or metastatic non-small cell lung cancer (NSCLC). Nivolumab as a monotherapy is registered for use in advanced renal cell carcinoma after the failure of prior therapy and in adult patients with recurrent or refractory classical Hodgkin’s lymphoma [14,17]. In Europe, the drug is also registered for the treatment of patients with recurrent or metastatic squamous cell carcinoma of the head and neck and in locally advanced or metastatic carcinoma of the urinary tract (bladder and urinary tract cancer) [14]. In the USA, nivolumab can also be used in the treatment of metastatic small-cell lung cancer after a failure of second-line chemotherapy and in the treatment of hepatocellular carcinoma after all other treatment options have been exhausted [13].

The effect of anti-PD-1, anti-PD-L1 or anti-CTLA-4 antibodies on immune processes is associated with the possibility of adverse effects (AE) related to the activity of the immune system, the infiltration of normal tissue by activated T lymphocytes and organ-specific autoimmune reactions which include, among others, pneumonia, colitis, hepatitis or endocrinopathies [1,18]. The mechanisms of these phenomena are poorly understood and require further research. For example, CTLA-4-deficient mice develop rapidly progressing lymphoproliferative disease with multi-organ T cell infiltration, ending in death at 3–4 weeks of life. This suggests that CTLA-4 takes part in the early phase of immune response. The lack of its expression results in a generalized autoimmune process [19]. Mice lacking PD-1 expression present delayed, organ-specific autoimmune diseases (e.g., arthritis, glomerulonephritis, cardiomyopathy of autoimmune etiology) [3]. This observation suggests that PD-1 is involved in the modulation of T lymphocyte activity during inflammatory processes on the periphery by limiting autoimmunity during antigenic stimulation, and its absence results in organ-specific auto-aggression [19,20]. In the case of pre-treatment auto-aggression, the PD-1 blockade usually leads to its exacerbation, but symptoms are reduced after symptomatic or substitution treatment and usually do not affect the possibility of continuing immunotherapy (this does not apply to serious autoimmune diseases such as lupus or systemic sclerosis) [14,21].

Data on the association of PD-L1 receptor expression on tumor cells and the risk of immune-related adverse events (irAE) are inconclusive. In the CheckMate-057 study, the group of patients with and without irAE did not differ from each other in terms of PD-L1 expression status [22]. Pembrolizumab-related studies provide similar data [23]. However, these reports are not conclusive due to the differences in the diagnostic methods used and the retrospective nature of the assessment. As for the assessment of the effectiveness of NSCLC treatment depending on the status of PD-L1 expression on tumor cells or on immune cells infiltrating tumor tissue, opinions are divided. Some studies suggest the greater efficacy of immunotherapy in patients with higher PD-L1 expression [9,22,24,25], but in the CheckMate-017 study of patients with squamous cell lung cancer, the same response to treatment was observed regardless of PD-L1 expression [26]. Other studies provide similar results [8,13].

It is likely that we do not yet know all the mechanisms responsible for the process of maintaining immunotolerance, but it is clear that the PD-1/PD-L1 system is one of the players responsible for maintaining this balance. Drugs affecting this mechanism by increasing the immunoreactivity of T lymphocytes against cancer cells disturb the state of immunotolerance. It is still unclear why the use of PD-1 or PD-L1 inhibitors mostly affects the function of the thyroid while CTLA-4 inhibitors cause pituitary disorders. The second question concerns the higher incidence of immunologic adverse effects of this therapy among women. This problem is broader, as autoimmune diseases generally affect women more often [3], which is explained by the influence of estrogen and prolactin, which have an immunomodulatory effect on the proliferation of autoreactive B lymphocytes in SLE (systemic lupus erythematosus) [3].

The frequency of significant adverse effects of nivolumab with a possible immune mechanism is estimated at 10–20% of patients undergoing immunotherapy [27], of which endocrine adverse events range from 2–8% [9,28] up to 23% [29]. Table 1 lists the frequency of endocrine AE of nivolumab with a possible immune background (data based on the analysis of 1994 patients receiving nivolumab monotherapy for all registered indications) [30]. As patients with a history of autoimmune disease were not included in the registration studies, the table also presents the occurrence of these adverse events according to other studies. In Poland, according to the guidelines of the National Health Fund treatment program, the presence of autoimmune diseases (except for type 1 diabetes, hypothyroidism treated with levothyroxine, albinism and psoriasis not requiring systemic treatment) is an exclusion criterion of nivolumab treatment [17].

## 4. Endocrine Adverse Effects Associated with Nivolumab Treatment

In patients with NSCLC (ChekMate-017 and CheckMate-057 studies), the incidence of hypothyroidism was 4–7%, while in the control group with docetaxel, no such cases were observed [22,26]. Other studies on this group of patients report hypothyroidism at a rate of 2.6–6.6% [35,36,43]. The incidence of hyperthyroidism and thyroiditis did not exceed 1.4% [22]. Of these patients, no more than 20% required immunomodulatory (steroid) therapy due to endocrine complications [22,26]. All these adverse effects were characterized as grade 1–2, and the time to onset (TTO) was on average 12.1 weeks (range 1.9–51.4 weeks). There were no cases of diabetes, hypophysitis or adrenal insufficiency in the study drug group [22,26]. Similar data on the incidence of endocrine complications were found in studies with another anti-PD-1 drug, pembrolizumab [23].

For comparison, immunotherapy-related pneumonia was reported at 3.1%, with a median time of onset of 3.5 months (range 1 day to 22.3 months), and the rate of immune-mediated hepatitis was 1.8% (3.3 months, range 6 days to 9 months), while immune-mediated skin adverse reactions showed a rate of 9% over 2.8 months (range 1 day to 25.8 months) [30].

## 5. Pituitary Gland Disorders

### 5.1. Epidemiology

Hypophysitis, which is connected with infiltration of the pituitary gland with immune cells—mainly lymphocytes and plasma cells [44]—in the course of immune checkpoint inhibitor therapy, is significantly more frequent in patients undergoing treatment with ipilimumab (1.8–17%) [30,44,45,46,47] than with drugs affecting the PD-1/PD-L1 (nivolumab—up to 0.6% [30,45,47], pembrolizumab—0.68% [45]). A metanalysis by Shang et al. did confirm the higher risk of pituitary gland inflammation among patients treated with PD-1 inhibitors compared to chemotherapy, everolimus or cetuximab [48]. Comparing the incidence of endocrinopathies in the course of treatment with nivolumab in combination with ipilimumab (combination therapy is registered in melanoma), rates of hypophysitis were 4.6–9% [13,30,45].

While primary lymphocytic pituitary inflammation is more often found in young women with childbearing potential, most often after delivery, in the case of ICI, treatment risk factors are old age and male gender [24].

### 5.2. Mechanisms

The higher risk of hypophysitis when treated with ipilimumab can be explained by the fact that anti CTLA-4 therapy can stimulate autoreactive T cells, as well as leading to the production of antibodies directed against pituitary antigens, infiltrating the gland tissue and increasing complement activation, which has been demonstrated in animal models [49]. In addition, CTLA-4 may be expressed on pituitary cells, thus becoming the target of anti-CTLA4 antibodies [49]. The expression of both RNA and CTLA-4 protein was found, particularly in lacto- and thyrotropic cells of the pituitary gland [49]. Tahir and co-workers delivered findings of the presence of auto-antibodies against guanine nucleotide-binding protein G (olf) subunit alpha (GNAL) and anti-integral membrane protein 2B (ITM2B) during ICI therapy-induced hypophysitis. The expression of these antigens was found in the normal pituitary glandular epithelium after immunohistochemical analysis [44]. However, the mechanisms underlying nivolumab-induced pituitary inflammation remain unknown [24].

### 5.3. Clinical Manifestations

The time of occurrence of pituitary inflammation during nivolumab monotherapy in the clinical trials ranged from 1.4 to 11 months (median 4.9 months), although cases of the development of pituitary insufficiency even 4 months after the cessation of nivolumab treatment were described [50]. In the case of combined therapy with nivolumab and ipilimumab, when endocrinopathies appear faster and may last longer [13,43,51], hypophysitis was described from 27 days to 5.5 months (median: 2.7 months) [30]. The clinical manifestation of pituitary inflammation is caused by the mass effect in the course of pituitary enlargement (headaches and visual disturbances such as diplopia, which are less frequent than in classic lymphocytic autoimmune hypophysitis), as well as a deficiency of tropic hormones leading to symptoms of peripheral gland failure of the thyroid, adrenals and gonads [52]. This does not always start with acute onset, which was explained by the low intensity of the immune response [53].

### 5.4. Diagnostics

In magnetic resonance imaging (MRI), we usually see a picture of small to moderate pituitary enlargement [54], most often with homogeneous enhancement after contrast, along with the thickening of the pituitary gland and subsequent atrophy of the gland and empty saddle syndrome [47,50,55]. During the acute phase of pituitary inflammation, the MRI picture may be normal in 25% of patients [24,56,57,58]. Sometimes, abnormalities in MRI may precede abnormalities in laboratory tests [47], and reverse situations have also been reported in which isolated adrenocorticotropin (ACTH) deficiency was accompanied by a normal MRI [59,60]. The resolution of inappropriate MRI features may be obtained 1–8 weeks after glucocorticoid treatment introduction [47].

In laboratory tests, the most common manifestation is ACTH and thyrotropin (TSH) deficiency, followed by hypogonadotropic hypogonadism and growth hormone (GH) and insulin-like growth factor-1 (IGF1) deficiency [45,55]. Regarding lactotropic cell function, both reduced and elevated prolactin (PRL) levels can be found [61]. Isolated cases of ACTH deficiency have also been reported [54,56,57,59,60,62]. Sekizaki et al. described a case of nivolumab-induced hypophysitis in which secondary adrenal insufficiency preceded a short phase of a high level of ACTH, with only slightly elevated cortisol levels. The authors explain these findings by the possibility of elevated ACTH as a result of the destruction of pituitary glands on the early stage or excretion of the biological inactive “big” ACTH [56]. Inactive ACTH excretion in lymphocytic hypophysitis was reported previously by other authors [63].

### 5.5. Treatment, Prognosis

Management and treatment methods are listed in Table 2. The healing process usually lasts from 1 to 8 weeks after starting steroid treatment [47]. Over time, a return to the normal functioning of the pituitary–thyroid axis has been reported, while gonadal and corticotropic line dysfunction usually persists, resulting in the need for long-term hormone replacement therapy [2,64], although few cases of recovery of gonadal as well as adrenal lines have been described [58,65]. In most cases, nivolumab therapy may be resumed, with most patients requiring continued long-term hormone replacement therapy [51].

## 6. Thyroid Gland Disorders

### 6.1. Epidemiology

Thyroid dysfunction in clinical trials of nivolumab affected a total of 11.7% of patients (Table 1) [30], and the risk of their occurrence was significantly higher than in the case of classical chemotherapy [69]. However, as the exclusion criteria in these studies included the presence of autoimmune diseases and, in practice, the presence of stable hypothyroidism treated with levothyroxine, including one with an autoimmune background, is not a contraindication for treatment [17], a higher incidence rate should be expected in real-world practice. This is reflected in other studies which mention the occurrence of thyroid disorders in the range of 3–23.3% [40,43,70,71,72,73], with a median time of onset of 81 days after starting treatment (with a range of 13 days to 6 months) [40]. The literature also points to the appearance of goiter at 0.3–0.8%, but with many potential causes of enlargement of the thyroid—both immunological and non-immunological—it is difficult to assess the risk of this complication in connection with checkpoint inhibitor treatment [40].

According to the metanalysis of Shang et al., PD-1 inhibitor treatment compared to anti-CTLA-4 therapy is related to a higher risk of thyroid gland disorders [48], similar to other authors [43]. The combination of these two molecules increases the risk of both hyper (8–12%) and hypothyroidism (up to 22%) [13,30,45,48]. The risk of this complication in the course of treatment with anti-PD-1 inhibitors and with anti PD-L1 inhibitors was comparable [43].

### 6.2. Clinical Manifestations

Disorders of the thyroid function in terms of the clinical picture include overt and subclinical hypothyroidism, overt and subclinical hyperthyroidism and thyroiditis with a wide range of manifestations, from asymptomatic with normal thyroid function to transient hyperthyroidism (thyrotoxicosis). Most often, however, destructive thyroiditis appears with the transient hyperactivity of the gland, without increased vascular flow in ultrasound, with negative thyroid stimulating antibodies and with the suppression of iodine uptake in scintigraphy (although this test is not routinely performed and may be difficult to interpret due to repeated radiological investigations with iodine contrast) [19,39]. In the course of destructive inflammation, after the thyrotoxicosis phase, most patients transition to hypothyroidism [41]. In addition, secondary hypothyroidism in the course of pituitary inflammation must be borne in mind, especially if the two consecutive TSH tests are below normal and free thyroid hormones are in the normal range or are reduced.

Destructive thyroiditis is a much more common cause of thyroid hormone excess than Graves’ disease [2,74], yet the differentiation of these two disorders is sometimes problematic. Elevated levels of anti-TSH antibodies are estimated to reach 3% in the patients treated with anti-PD-1 [9]; on the other hand, in described cases of nivolumab-induced Graves’ disease, anti-TSH antibodies were negative (the diagnosis was based on the enlargement of glands, hypervascularity at Doppler and increased and diffuse uptake of I131 in scintigram) [74]. Thyroid function disturbances in the course of checkpoint inhibitor treatment usually develop 2–4 weeks after drug administration. Data concerning nivolumab monotherapy are presented in Table 1. In the case of combined therapy with nivolumab and ipilimumab, these disorders develop earlier [51]. In patients with thyroid disease prior to therapy, its exacerbation is possible, although not in all cases [4,19,21,33,75,76]. Patients with subclinical thyroid disorders were observed in whom in the course of immunotherapy did not progress to overt [40].

Single cases of orbitopathy (exophthalmos, burning eyes, photophobia) in the course of ipilimumab treatment have been reported [77]. This complication during treatment with nivolumab is very rare; to date, only one case has been described [78].

### 6.3. Mechanisms

Studies on the pathogenesis of endocrine complications in PD-1 inhibitor therapies are lacking [19,79]. Yamauchi et al. showed—with RT-PCR techniques and with immunoblotting—the expression of PD-L1 and PD-L2 in healthy thyroid tissue (as well as in cell line HTC/C3 of undifferentiated thyroid cancer) [41]. PD-L2 expression in thyroid tissue was shown to be relatively high; thus, it seems logical that the blockade of the PD-1 signal pathway can exacerbate autoaggression in healthy tissue with PD-L1 and PD-L2 expression [41]. The studies on mouse models show that the PD-1 blockade activates lymphocytes B and increases the role of humoral mechanisms of the response [19]. However, in thyroiditis caused by anti-PD-1 treatment, a cellular (T-cell-mediated) rather than humoral (antibody-mediated) mechanism of immune response is observed, which plays a major role in its pathogenesis [19]. Increased monocyte activation is also observed. The total numbers of circulating lymphocytes T and B, monocytes, granulocytes and dendric cells remained unchanged. In flow cytometry, differences in subpopulations of lymphocytes T and other immunocompetent cells were observed [19]. In patients with thyroiditis who were treated with anti-PD-1 inhibitors, diminished numbers of immature NK cells (natural killer cells) (CD56brCD16-) as well as of immunosuppressive monocytes with low expression of HLA-DR (human leukocyte antigen–DR isotype) (CD14+HLA-DRlo/neg) were documented [19]. Monocyte activation through the upregulation of HLA-DR could be a possible mechanism of this disturbance [19]. Tumor epitopes can have a similar amino acid sequence with thyroid antigens, and the cross-presentation of these epitopes on HLA molecules may be involved in the thyroid irAE mechanism [80].

The role of thyroid antibodies in the pathogenesis of thyroid lesions due to anti-PD-1 treatment remains unclear. In some studies, thyroid antibodies were present in the majority of patients with abnormal thyroid function in the course of anti-PD-1 treatment [70]. However, there have also been studies in which the presence of the antibodies was not common [19,58,73,80]; for example, in the study by Gridelli, a lack of antibodies was found even in one third of the patients [9], and according to Peiró’s analysis, autoimmunity was found only in one third of patients with thyroid disorders due to nivolumab treatment [72]. These observations suggest a complex mechanism of irAE in the thyroid, including an antibody-independent mechanism of cellular response or the role of other thyroid antibodies which are not evaluated routinely. Besides this, the PD-1 blockade affects mainly the pathway related to lymphocytes T. However, as PD-1 receptors are also present on activated lymphocytes B, immunotherapy also influences the humoral pathway of the immune response [70]. It remains to be determined whether the appearance of thyroid antibodies in the course of the treatment causes thyroid inflammation or if it is rather the result of the humoral response to the presence of thyroid antigens in the course of gland destruction [19]. The antibodies appear soon after anti-PD-1 drug administration, suggesting that the therapy probably unveils thyroid inflammation which already existed prior to the therapy but in a latent form [70]. The analysis of the problem is also complicated by the fact that Hashimoto’s disease affects approximately 4% of the general population and that thyroid antibodies are detected in 11% of healthy individuals [70]. However, an elevated level of thyroid antibodies found at the beginning of the treatment is a risk factor of both the development as well as exacerbation of thyroid disorders in the course of anti-PD-1 treatment [19,21,40,75,81,82]. In order to determine the potential differences in the pathogenesis of ICI and autoimmune thyroid disease, further investigations are needed [19].

### 6.4. Diagnostics

Hypothyroidism is defined as elevated TSH with normal FT4 and/or FT3 levels. In the case of hyperthyroidism, decreased TSH concentration together with an elevated or normal free thyroid hormone is observed. Thyroiditis/thyreotoxicosis is defined by the presence of a reduced level of TSH with normal or elevated free thyroxine (FT4) or free triiodothyronine (FT3) that spontaneously resolved or converted into hypothyroidism. Assessment of thyroid antibodies—anti-thyroid peroxidase antibodies (aTPO) and antithyroglobulin antibodies (aTG)—and, in the case of hyperthyroidism, anti-TSH antibodies, is needed.

In the course of anti-PD-1 treatment, the enhanced and disseminated uptake of fluorodeoxyglucose (FDG) in PET-CT (positron emission tomography) is particularly common for destructive thyroiditis (less common for Graves’ disease). It is worth mentioning that, in patients who present this thyroid image (increased thyroid uptake of FDG-PET, defined as SUVmax > 4.0) before treatment introduction, thyroid dysfunction in the course of the therapy should be expected [19,83].

### 6.5. Treatment, Prognosis

The management algorithm for thyroid function disorders is presented in Table 2. In most cases, only symptomatic treatment is recommended [13,30,51].

In nivolumab-induced hypothyroidism, the normalization of thyroid function is possible. In the nivolumab registration studies, a return of euthyroidism was observed in 35% of cases where the drug was administered in monotherapy [30]. For comparison, in post-partum thyroiditis, permanent hypothyroidism affects 54% of women [84]. In the case of hyperthyroidism, in nivolumab monotherapy (all indications), a return to euthyroidism was observed in 76% of cases [13,30]. There are studies which suggest a protective role of glucocorticoid therapy in the thyrotoxicosis phase against permanent hypothyroidism after nivolumab therapy; however, they are based on small groups of patients, and thus further observation is required [19,41].

## 7. Disorders of Adrenal Glands

### 7.1. Epidemiology

Secondary adrenal insufficiency in the course of hypophysitis (with a higher risk in the course of CTLA-4 inhibitor treatment) or due to metastatic lesions in the pituitary have to be taken into consideration. Primary adrenal insufficiency in the registration studies was described in 1% of cases [29]. Although in the studies concerning lung cancer (CheckMate 017 and 057), this complication was not reported [22,26], in AdbelRahman metanalysis, its relative risk was increased, with RR 3.87 (95% confidence interval (CI): 1.12–13.41) [69]. In the case of nivolumab treatment in other studies, this side-effect affected 0.85–1.9% of lung cancer patients [31,36] and 1.9% of renal cancer patients [37], whereas in melanoma malignum, this complication was estimated to affect even 3–6.5% [42,85], with this higher proportion probably being due to former ipilimumab treatment. Based on Barosso–Sousa metanalysis, incidence of primary adrenal insufficiency in the course of ICI therapy was found for 0.7% of patients [43]. However, in combined treatment, this proportion was as high as 4.2% [43].

### 7.2. Clinical Manifestations

The clinical features of adrenal insufficiency is variable, depending on whether the onset is chronic, with nonspecific symptoms (fatigue, low blood pressure, orthostatic hypotension, weight loss, hyperpigmentation) or acute, leading to adrenal crisis with peripheral vascular collapse [86,87]. Diagnosis is often very difficult in patients with cancer. It is crucial to inform the patient and his treatment team about this possible life-threatening complication.

### 7.3. Diagnostics

Hormonal assessment involves serum or salivary cortisol levels, as well as urinary cortisol excretion (low values) and the plasma corticotropin (ACTH) (decreased or low normal values in the case of secondary and elevated values in the case of primary adrenal insufficiency). Additionally, in laboratory tests, hypoglycemia, hyponatremia and hyperkalemia can be noted [86,87].

In radiologic image studies, the adrenals are enlarged [2]. In PET-CT (performed for oncologic reasons), bilateral enhanced FDP uptake may indicate autoimmune adrenalitis, suggesting the need for adrenal function evaluation even when the symptoms of adrenal insufficiency are poor [86].

### 7.4. Treatment, Prognosis

The management algorithm for adrenal insufficiency is presented in Table 2.

Owing to the fact that adrenal insufficiency manifests when the majority of the adrenal cortex is destroyed, the normalization of adrenal function after immunotherapy should not be expected [87].

## 8. Diabetes

### 8.1. Epidemiology

Type 1 diabetes is a rare complication of PD-1 treatment (Table 1) affecting no more than 1–1.5% of cases. Based on Barosso–Sousa metanalysis, the incidence of this side-effect for patients treated with ICI affects 0.2%, and this complication reached grade 3 or more in only 0.1% of cases [43]. In the analysis of Kotwal et al., which involved 1444 patients treated with ICI (anti-CTLA4, anti-PD-1, anti-PD-L1), new onset diabetes was found in 0.8% of patients, and 0.6% experienced a worsening of pre-existing diabetes. Taking patients treated with nivolumab into consideration, these values were 0.2% and 0.8%, respectively [88]. Considering treatment with ICI in the course of anti PD-1/PD-L1 therapy, the incidence of type 1 diabetes is more common than in the course of ipilimumab therapy [51].

### 8.2. Clinical Manifestations

Classic clinical features involve chronic polydipsia, polyuria and weight loss with hyperglycemia and ketonuria. The time of occurrence is on average 4.4 months after the introduction of therapy (ranging from 15 days to 22 months) [30,89]. The development of diabetes in the state of ketoacidosis after two doses of nivolumab was described [27]. Clinically, immunotherapy-induced type 1 diabetes is characterized by a short time interval from the introduction of treatment to diabetic symptoms including ketoacidosis [7,39,90]. This problem is more common in the Japanese population [90]. In contrast to typical type 1 diabetes mellitus, a remission phase is not observed [24]. The retrospective analysis of plasma collected before immune therapy introduction often confirms the presence of the auto-antibodies but with the levels of insulin, c-peptide and glucose within normal ranges, suggesting the presence of the disease process in the latent phase [27,91].

### 8.3. Mechanisms

The patomechanism of type 1 diabetes mellitus development in anti-PD-1 treatment is not yet fully understood. The presence of antibodies against insulin, islet cell and antiglutamic acid decarboxylase enzyme has been found [7,27,39,92,93]; however, the inhibition of the PD-1/PD-L1 signaling system increases the tendency for organ infiltration by autoreactive regulatory lymphocytes T and the destruction of the pancreas, suggesting a cellular mechanism of the pathology [2], with the phenomenon being confirmed in mouse models [51]. The inhibition of the PD-1 or PD-L1 system aggravates diabetes in female individuals with prediabetes (mouse model). PD-L1 expression in pancreatic islets explains the exacerbation of diabetes in the course of treatment with anti-PD-L1 antibodies [3]. The roles of genetic factors and of the HLA system must also be considered. Arauja et al. suggest a higher risk of diabetes development in the course of anti-PD-1 treatment in individuals with genotype DR3-DQ2 and DR4-DQ8 HLA-II (DRB1*03-DQB1*02:01 and DRB1*04-DQB1*03:02) [94]. According to Lowe, this risk is higher in the case of HLA-I A2 and HLA-II DQB1*060 genotypes [93]. In the analysis of Kotwal patients experiencing diabetes, they had higher single-nucleotide polymorphisms in the PD-1 gene [88]. A recent systematic review identified 91 patients with diabetes that appeared during ICI treatment (anti-PD-1 or anti-PD-L1 as monotherapy or in combination with ipilimumab). In this group, 71% of patients experienced diabetic ketoacidosis; in 84%, the level of C-peptide was low, and half of the group presented elevated lipase levels (52%). The presence of at least one type of autoantibody was found in 53% (anti-islet cell antibodies, anti-glutamic acid decarboxylase autoantibodies, insulinoma-associated antigen-2 (IA-2) autoantibodies, anti-insulin antibodies, zinc transporter 8 autoantibodies). The initial evaluation of the diabetes-specific auto-antibodies could reveal predisposed patients; however, considering the low incidence of this complication, it is not a routine procedure [91].

### 8.4. Diagnostics

Diagnosis is made based on universal criteria for diabetes [95]. Patients undergoing nivolumab treatment should be carefully monitored for elevated levels of blood sugar. Antibodies against islet cell (ICA), against glutamic acid decarboxylase (GAD), anti-insulin antibodies and C-peptide should be measured in order to distinguish between type 1 and type 2 diabetes [92].

There are reports of diffusely increased FDG uptake on PET-CT scans in the pancreas of patients with ICI-induced type 1 diabetes found at diagnosis [88].

### 8.5. Treatment, Prognosis

In type 1 diabetes onset in the course of anti-PD-1 therapy, anti-diabetic treatment has to be undertaken immediately. This is based on general rules, including first constant intravenous insulin infusion, which is then followed by intensive subcutaneous insulin therapy. It also includes fluid and electrolyte supplementation [89]. After the normalization of glucose metabolism, ICI treatment can be undertaken again [30,51]. Whether high doses of glucocorticoids could prevent the complete loss of beta cell function in these patients remains to be determined. Therefore, glucocorticoid therapy is not recommended. Besides the above, in the course of such a treatment, a worsening of glycemic control can be expected [51]. There are no data on the remission of the disease after the termination of the immunotherapy. The possibility of diabetes onset in the course of treatment implies the need for regular glycemia control in patients in the course of immunotherapy [91].

## 9. Management of Adverse Events during Nivolumab Treatment

In the case of endocrine side-effects, drug-dose modification is recommended (according to Table 2) [13,14,30], and if necessary, symptomatic treatment should be initiated, including systemic glucocorticoid therapy [30]. Usually, immunologic complications have a mild to moderate grade. The incidence of higher grade irAE (grades 3–4) does not exceed 4%. Of these, the most common are skin symptoms and gastrointestinal tract-related and endocrine-related problems [96].

Usually, management of thyroid function disorders includes only observation, without treatment introduction. This is true particularly in the case of hyperthyroidism. All patients with overt hypothyroidism and patients with subclinical hypothyroidism who report fatigue or other symptoms typical for hypothyroidism should have levothyroxine treatment introduced [51,67]. In some studies, levothyroxin supplementation is recommended when TSH exceeds 10 [67,70]. Glucocorticoids are not recommended [30]. In a study with 1994 patients receiving nivolumab in whom hypothyroidism developed, 79% of them needed levothyroxine treatment, whereas in 4%, systemic glucocorticoid therapy was administered. Thyroid function normalization was observed in 35% of the individuals [13,30].

In hyperthyroidism, usually symptomatic treatment is sufficient. According to nivolumab registration studies, in patients in whom hyperthyroidism developed, 39% received an anti-thyroid drug whereas 9% were treated with glucocorticoids. Normalization of thyroid function was observed in 76% of patients [13,30]. It seems that, in patients with NSCLC in whom chronic TSH decline is observed and there are mild symptoms of subclinical hyperthyroidism, anti-thyroid drug administration could be beneficial [9].

In the majority of complications at grades 3 or 4, temporary nivolumab discontinuation and systemic glucocorticoid therapy ameliorate or reverse the symptoms. According to nivolumab registration studies, endocrine adverse effects demanding temporary or permanent treatment discontinuation did not exceed 1%—in the case of hypophysitis, 0.2% and 0.1%, respectively, and concerning adrenal insufficiency, 0.5% and 0.1%. There was no need for the discontinuation of ICI treatment because of thyroid disorders or diabetes [13,30]. Among those patients, glucocorticoid therapy (anti-inflammatory doses) was introduced in 33% of patients with hypohysitis, 25% with adrenal insufficiency, 4% of patients with hypothyroidism and 9% of patients with hyperthyroidism [13,30]. In the case of glucocorticoid therapy introduced for symptomatic hyperthyroidism due to destructive thyroiditis, when a prednisolone equivalent of 10 mg/day for more 12 weeks is needed, it is usually an indication of the need for permanent immunotherapy discontinuation [30].

## 10. IrAE and Efficacy of Immunotherapy

Analyzing immunologic adverse effects in terms of cancer prognosis, there are data showing higher treatment effectiveness (higher objective response rate, ORR) in patients with irAE in comparison to the patients in whom there were no such complications [96]. In the case of more than one immune complication, the prognosis is better than in the case of only one immune side-effect [96,97,98,99]. The onset of vitiligo in patients with melanoma malignum treated with immunotherapy was related to a higher survival rate [98]. In lung cancer patients, irAE development or the presence of anti-thyroid antibodies before the treatment introduction were independent prognostic factors of positive response to the therapy [21]. Furthermore, irAE prolongs the time to next treatment or death (TTNTD) [100]. Further studies report a relationship between skin [98,100] and endocrine irAE with progression free survival (PFS) prolongation, the presence of any irAE and skin irAE with overall survival (OS) prolongation [100]. In the study by Osorio et al., OS prolongation was confirmed in patients with NSCLC treated with ICI in whom thyroid immune complications developed [70]. Similar results were also obtained by other authors [4,72,101,102,103]. In the study by Yamanuci et al., a significantly longer PFS was confirmed among patients with thyroid irAE compared with those without such side effects; this was found in NSCLC patients but not among melanoma patients [83]. The authors also concluded that overt thyroid irAEs have a stronger impact than subclinical thyroid irAEs [83]. In the metaanalysis performed by Zhou et al. the occurrence of endocrine, as well as dermatological irAEs, was connected with significantly better OS and PFS among patients undergoing immunotherapy. This finding was related to patients treated with ICIs as a monotherapy but not combination therapy [104]. Besides the above, a favorable influence on survival was observed in patients treated with PD-1 inhibitors but not those treated with CTLA-4 inhibitors [104]. Better prognosis was confirmed in early irAE [100] or low-grade irAE [29,104]. IrAE development can be related to enhanced immunologic response against neoplasm tissue. Serious adverse effects (at grades 3–4) usually concern small groups of patients; thus, achieving statistical significance is problematic. Furthermore, high-grade irAE is usually related to higher morbidity because of the complication itself. However, not all studies confirm this correlation [73,96]. The detailed mechanism responsible for better disease prognosis in irAE patients remains unclear [100] and needs further study.

## 11. Conclusions

In the management of patients receiving immunotherapy, awareness of the possibility of irAE which are different from AE of classic chemotherapy is crucial. Both the patient and treatment team (including the general practitioner) should be monitoring for their possible symptoms. Many of the irAE are linked to the endocrine system. The risk of hypophysitis is significantly lower in nivolumab therapy in comparison to anti-CTLA-4 treatment. Thyroid disorders (both hypo and hyperthyroidism) are more common in nivolumab patients. As the severity of the thyroid adverse effects rarely exceeds grade 2, the control of TSH and Ft4 every 2–4 weeks is usually sufficient [9,51]. Primary and secondary adrenal insufficiency are possible complications demanding immediate treatment. Before anti-PD-1 treatment introduction, an evaluation of the patient for autoimmune diseases should be performed. Individuals in whom autoimmune diseases are diagnosed prior to immunotherapy are at risk of its exacerbation. Thyroid antibodies and type 1 diabetes-related antibodies are considered risk factors and therefore—when detected—organ-specific immune complications should be expected. IrAE development in the course of nivolumab treatment may be related to better responses to the therapy in patients with NSCLC. However, this matter needs further investigation.

## Figures and Tables

**Table 1:** Endocrine adverse effects of nivolumab treatment.

Adverse Event	Occurrence Rate (%) [30]	Median Time of Occurrence of Adverse Event [30] (Months)	Frequency of Occurrence Observed in Other Studies
Hypophysitis	0.6	4.9 (range 1.4 to 11 months)	0.5–0.9 [31,32,33,34]
Adrenal Insufficiency	1	4.3 (range 15 days to 21 months)	0.85–1.9 [35,36,37]
Type 1 Diabetes Mellitus	0.9 ^1^	4.4 (range 15 days to 22 months)	0.1–1.5 [32,37,38,39]
Hypothyroidism/Thyroiditis	9	2.9 (range 1 day to 16.6 months)	2.6–10.1 [33,34,35,36,40,41,42]
Hyperthyroidism	2.7	1.5 (range 1 day to 14.2 months)	0.8–15.3 [34,35,37,40,41,42]

^1^ including two cases of diabetic ketoacidosis [30].

**Table 2:** Adapted management algorithm for endocrine adverse events during nivolumab treatment [9,13,30,51,66,67].

Adverse Events	Intensity [14,68]	Recommended Nivolumab Dosage Modification	Monitoring/Treatment
Hypophysitis	**Grade 1**—asymptomatic or mild symptoms, e.g., fatigue, weakness, loss of appetite of mild intensity, no headache	Continued treatment	-Hormonal evaluation -Pituitary MRI -Clinical and hormonal evaluation every 1–3 weeks -Repeat MRI after one month if the symptoms remain and the hormonal tests and/or MRI are normal -Hormonal substitution if necessary (e.g., hydrocortisone 10 to 20 mg orally in the morning, 5 to 10 mg orally in early afternoon; levothyroxine by weight, testosterone or estrogen therapy as needed in those without contraindications. Always start corticosteroids several days before thyroid hormone to prevent precipitating adrenal crisis. Follow FT4 for thyroid hormone replacement titration (TSH is not accurate). ≥Grade 2: 1 mg/kg/day prednisone/prednisone equivalents -Substitution treatment if necessary -Analgesic treatment in case of headaches -In case of clinical improvement (with or without substitution treatment continuation), taper steroids over at least 1 month before resuming treatment -Return to treatment in case of return of AE to level 0–1
	**Grade 2**—moderate symptoms, e.g., headaches without vision disturbances, fatigue, worse well-being, hemodynamically stable, no electrolyte abnormalities	Withhold dose, temporary drug interruption	
	**Grade 3**—serious mass-effect symptoms, e.g., headaches, vision disturbances or severe symptoms of adrenal insufficiency	Withhold dose, temporary drug interruption	
	**Grade 4**	Permanently discontinue	
Adrenal insufficiency	**Grade 1** Asymptomatic;	Clinical or diagnostic observations only; no intervention in terms of nivolumab treatment	-Clinical and hormonal evaluation repeated every 1–3 weeks -Glucocorticoids administered in the substitutional doses (hydrocortisone: 10 to 20 mg orally in the morning, 5 to 10 mg orally in early afternoon or prednisone: 5 to 10 mg daily, fludrocortisone (0.1 mg/d) for mineralocorticoid replacement in primary adrenal insufficiency -Grades 3–4: 1 to 2 mg/kg/day prednisone equivalents followed by corticosteroid taper over at least 1 month -Return to treatment in case of return of AE to level 0–1
	**Grade 2** Moderate symptoms;	Consider withholding dose	
	**Grade 3** Severe symptoms;	Discontinuation of treatment/hospitalization	
	**Grade 4** Life-threatening symptoms		
Type 1 diabetes	**Grade 1** Asymptomatic or mild symptoms; fasting glucose value >ULN-160 mg/dl (>ULN-8.9 mmol/L), no evidence of ketosis	Clinical or diagnostic observations only; no intervention in terms of nivolumab treatment	-Monitoring and treatment of hyperglycemia -In case of AE returning to level 0–1, return to treatment
	**Grade 2** Moderate symptoms, fasting glucose value >160–250 mg/dl (>8.9–13.9 mmol/L)		
	**Grade 3** Fasting glucose value >250–500 mg/dl (>13.9–27.8 mmol/l)	Withhold dose, hospitalization indicated	
	**Grade 4** Fasting glucose value >500 mg/dl (>27.8 mmol/L)	Discontinuation of treatment, hospitalization indicated	
Thyroid disorders-Hypothyroidism	**Grade 1** Asymptomatic or few symptoms TSH elevated (<10 mUL/L), FT3, FT4-normal aTPO and anti-TG usually high	There are no recommended modifications of dosage of nivolumab. In severe cases, (Grade ≥ 3) consider withholding ICI	-Monitor thyroid function prior to and periodically during treatment (before each cycle) -Levothyroxine in case of hypothyroidism -Grade ≥ 3: Hospitalization Supportive therapy for severe cardio-respiratory symptoms Return to Grade 2—consider return to immunotherapy
	**Grade 2** Mild symptoms (fatigue, weight gain, constipations, dry skin, eyelid edema, puffy face) Low FT3 and/or FT4, TSH > 10 mUI/L aTPO/antiTg usually high		
	**Grade 3–4** Moderate–severe symptoms (bradycardia, hypotension, pericardial effusion, depression, hypoventilation, stupor, lethargy to myxedema coma) -Very low FT4, FT3 -TSH very high -aTPO and antiTG usually high		
Thyroid disorders Hyperthyroidism	**Grade 1** Asymptomatic -FT3, FT4 normal -TSH suppressed (<0.1 mUI/L), antiTPO/aTG normal or high	There are no recommended modifications of dosage of nivolumab. In severe cases (Grade ≥ 3), consider withholding ICI	-TSH, FT4, FT3 before each cycle -Close monitoring of thyroid function to catch transition to hypothyroidism in patients with thyroiditis and hyperthyroidism -Overt hyperthyroidism: introduce beta-blocker (propranolol/atenolol/metoprolol) -Consider glucocorticoid therapy—prednisone 1 to 2 mg/kg/d or equivalent tapered over 1 to 2 weeks (not routinely) -Anti-thyroid drug (thiamazol/PTU) in case of Graves’ disease -Grade ≥ 3: Hospitalization -In case of destructive thyroiditis, consider treatment with oral glucocorticoid (prednisone equivalent 0.5–1 mg/kg/day followed by dose reduction) -In case of withholding ICI treatment, consider restarting when symptoms controlled
	**Grade 2** Mild symptoms: weight loss, increased appetite, anxiety and irritability, muscle weakness -TSH suppressed (<0.1 mUI/L) -FT4, FT4 high -aTPO/aTG normal or high -TSAbs high in case of Graves’ disease		
	**Grade 3–4** Moderate to severe symptoms: arrhythmia, atrial fibrillation, tremor -TSH suppressed (<0.1 mUI/L) -FT4, FT4 high -aTPO/aTG normal or high -TSAbs high in case of Graves’ disease		

Grade 1—Mild: asymptomatic or mild symptoms; clinical or diagnostics observations only, intervention not indicated; Grade 2—Moderate: symptomatic, limiting age appropriate instrumental ADL *. Minimal, local or noninvasive intervention indicated; Grade 3—Severe: medically significant but not immediately life-threatening; hospitalization or prolongation of existing hospitalization indicated; disabling; limiting self-care ADL **; Grade 4—Life-threatening consequences: urgent intervention indicated; Grade 5—Death related to AE. * ADL—Activities of daily living; Instrumental ADL refers to preparing meals, shopping for groceries or clothes, using the telephone, managing money, etc.; ** Self-care ADL refers to bathing, dressing and undressing, feeding self, using the toilet, taking medications and not being bedridden. ULN—Upper limit of normal; TSH—thyrotropin; aTPO—anti-thyroid peroxidase antibodies; aTG—antithyroglobulin antibodies; TSAbs—thyroid stimulating antibodies.
